# PI3K-AKT Pathway Modulation by Thymoquinone Limits Tumor Growth and Glycolytic Metabolism in Colorectal Cancer

**DOI:** 10.3390/ijms23042305

**Published:** 2022-02-19

**Authors:** Shahid Karim, Abdulhadi S. Burzangi, Aftab Ahmad, Nasir Ali Siddiqui, Ibrahim M. Ibrahim, Priyanka Sharma, Walaa A. Abualsunun, Gamal A. Gabr

**Affiliations:** 1 Department of Pharmacology, Faculty of Medicine, King Abdulaziz University, Jeddah 21589, Saudi Arabia; burzangi@kau.edu.sa (A.S.B.); imibrahim1@kau.edu.sa (I.M.I.); 2 Health Information Technology Department, Faculty of Applied Studies, King Abdulaziz University, Jeddah 21589, Saudi Arabia; abdulsalam@kau.edu.sa; 3 Department of Pharmacognosy, College of Pharmacy, King Saud University, Riyadh 11451, Saudi Arabia; nasiratksu@gmail.com; 4 Center for Innovation in Personalized Medicine, King Fahad Medical Research, King Abdulaziz University, Jeddah 21589, Saudi Arabia; aamrahs@kau.edu.sa; 5 Department of Pharmaceutics, Faculty of Pharmacy, King Abdulaziz University, Jeddah 21589, Saudi Arabia; wabuassonon@kau.edu.sa; 6 Department of Pharmacology and Toxicology, College of Pharmacy, Prince Sattam Bin Abdulaziz University, Al-Kharj 11942, Saudi Arabia; g.gabr@psau.edu.sa; 7 Agricultural Genetic Engineering Research Institute, Agriculture Research Center, Giza 12619, Egypt

**Keywords:** thymoquinone, Warburg effect, antimetabolite, colorectal cancer

## Abstract

Colorectal cancer (CRC) is the third leading cause of death in men and the fourth in women worldwide and is characterized by deranged cellular energetics. Thymoquinone, an active component from Nigella sativa, has been extensively studied against cancer, however, its role in affecting deregulated cancer metabolism is largely unknown. Further, the phosphoinositide 3-kinase (PI3K) pathway is one of the most activated pathways in cancer and its activation is central to most deregulated metabolic pathways for supporting the anabolic needs of growing cancer cells. Herein, we provide evidence that thymoquinone inhibits glycolytic metabolism (Warburg effect) in colorectal cancer cell lines. Further, we show that such an abrogation of deranged cell metabolism was due, at least in part, to the inhibition of the rate-limiting glycolytic enzyme, Hexokinase 2 (HK2), via modulating the PI3/AKT axis. While overexpression of HK2 showed that it is essential for fueling glycolytic metabolism as well as sustaining tumorigenicity, its pharmacologic and/or genetic inhibition led to a reduction in the observed effects. The results decipher HK2 mediated inhibitory effects of thymoquinone in modulating its glycolytic metabolism and antitumor effects. In conclusion, we provide evidence of metabolic perturbation by thymoquinone in CRC cells, highlighting its potential to be used/repurposed as an antimetabolite drug, though the latter needs further validation utilizing other suitable cell and/or preclinical animal models.

## 1. Introduction

Tumor cells are supported by metabolic rewiring in their growth, proliferation, survival, maintenance and reprogram the pathways of nutrient acquisition to meet the bioenergetics [1]. This metabolic reprogramming is known as one of the prominent hallmarks of almost all cancers [2]. The hypothesis that cancer cells uptake glucose and produce a significant amount of lactate in the presence of normoxia due to weak mitochondrial function led to the broadly held delusion that cancer cells follow glycolysis as their major source of ATP [3]. Many tumor cells reside in nutrient and oxygen-deficient environments, which makes a heterogeneous build-up that led cancer cells to adopt several methods to sustain mitochondrial function for survival [4]. Metabolism is improved in differentiated non-dividing cells to supply ATP through OXPHOS. Cells that proliferate at faster rates whether tumor cells or normal embryonic, immune and regenerating cells need ATP as well as anabolic constructive material for biomass improvement and nutrient refilling [5,6]. A glycolytic fermentative constitution similar to tumor cells is displayed by highly proliferative normal cells as well; a major division in cancer cells is the loss of the regulatory response kink [7]. This exclusive glucose-dependent phenotype together with enhanced lactate tumors serving as potential biomarkers of poor patient survival has motivated us along with other scientists [8,9,10,11] to disengage glycolysis in cancers as a presumed therapeutic move. 

In human cancers, the most commonly activated pathway is said to be the phosphoinositide 3-kinase PI3K-AKT pathway [12]. This pathway renovated cellular metabolism in cancer cells by increasing the action of nutrient transporters and metabolic enzymes by oncogenic activation following the anabolic demands of abnormally growing cells. It was reported that the PI3K pathway controls the reprogramming of hexokinases (HKs), the enzymes which play a potent role in the conversion of glucose into glucose-6-phosphate (a major rate-limiting stage of glycolysis) and other metabolic pathways which lead to the up-regulation of HK II expression [13,14]. 

In glucose metabolism, the catalysis loyalty is started by Hexokinases (HKs). Glucose transporters (GLUTs) transport glucose via the plasma membrane and are phosphorylated by Hexokinases to produce glucose-6-phosphate [15]. In humans, four isoforms of Hexokinases were categorized which are HK: I, II, III and IV. Being structurally similar, the four isoforms vary in expression pattern, cellular localization and properties in regulation. The dominantly expressed among these is HK II which is found to be up-regulated in most of the tissues [16]. Hexokinase II was found to be over-expressed in most cancers leading to enhanced glycolytic rate, which is a phenotype for cancer cells [17]. Many studies have suggested HK II as a therapeutic target for treating cancer [18,19]. Therefore, it is important to evaluate compounds/drugs that could be effective in repressing HK II and hence could inhibit tumor metabolism.

Thymoquinone (TQ), also known as black seed or black cumin, is a key bioactive phytochemical constituent of Nigella sativa and it plays a major role in providing health benefits via anti-oxidant, anti-microbial, anti-diabetic, anti-inflammatory effects, metabolic syndrome disorders and has a long history of medicinal use in traditional medicinal practices [20,21,22,23]. Many studies have established TQ as a potential anti-cancer agent by inhibiting proliferation, migration, invasion and angiogenesis in a variety of cancer cells [24]. Thymoquinone targets cancer signaling molecules and pathways through different axes in various cancers. In prostate cancer thymoquinone targets via the down-regulation of the androgen receptor (AR) and the proliferation regulator E2F-1 [25]; the (STAT3) pathway in human multiple myeloma cells [26]; JAK2 and c-Src in gastric cancer cells [27]; Bax up-regulation, Bcl-2 inhibition and activation of caspases in colorectal cancer [28]; inhibiting p38 MAPK in oral cancer [29]; activating p73 and UHRF1-dependent mitochondrial and cell cycle checkpoints in acute lymphoblastic leukemia [30,31], and the inhibition of the Notch signaling pathway in hepatic carcinoma [32]. On the other hand, the effect of thymoquinone on cancer metabolism, an emerging hallmark of cancer, is unclear. 

In the present study, we investigated the effect of thymoquinone in modulating PI3K-AKT/HK2-mediated deregulated cellular energetics in colorectal cancer (CRC) cells. We report for the first time that TQ inhibits HK2-mediated glycolytic metabolism, otherwise necessary to fuel the proliferation, clonogenicity and metastatic predisposition of CRC cells. Taken together, the data from the present study reveal the novel anticancerous potential of TQ by modulating cellular energetics, at least in part, via the PI3K-AKT/HK2 pathway.

## 2. Results

### 2.1. Thymoquinone Induces Cell Death in CRC Cells

**Figure 1:**
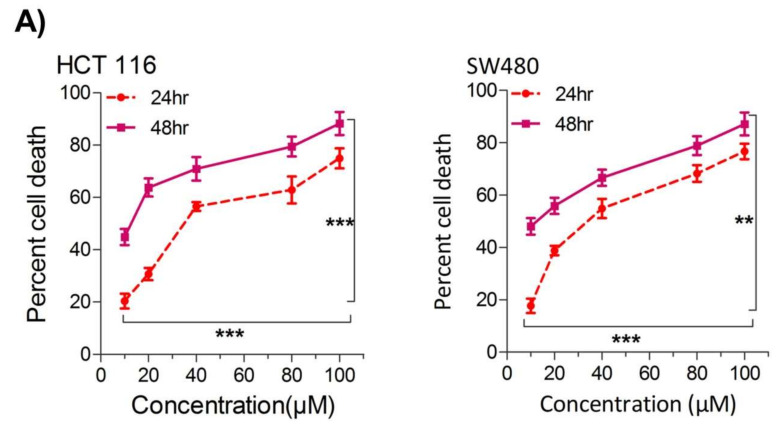

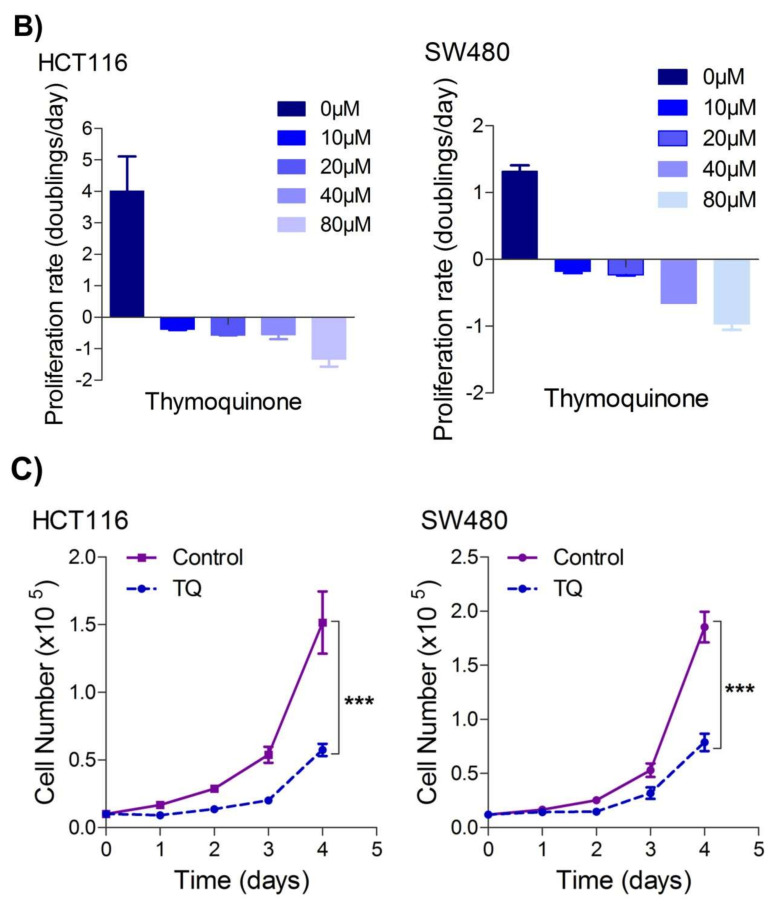
Thymoquinone induces cell death and hampers cell proliferation in CRC cells. HCT116 and SW480 CRC cells were treated with thymoquinone as indicated. (**A**) Thymoquinone induced cell death in a dose and time-dependent manner, statistical significance was calculated by two-way ANOVA, considering both time and concentration-dependent observations. (**B**) Thymoquinone treatment-induced cell death/inhibited cell doublings per day over a time period of 1–4 days. (**C**) Pre-treatment of thymoquinone to HCT116 (21.71 µM) and SW480 (20.53 µM) led to a significant reduction in cell proliferation over time (0–4 days), statistical significance was calculated by employing two way ANOVA following appropriate post hoc test (Bonferroni test). The results are expressed mean ± SEM (*n* = 3 or 4). ** *p* < 0.01, *** *p* < 0.001.

Our first aim was to study the effect of thymoquinone on the growth inhibition against two human colorectal cancer cells lines, HCT116 and SW480. It was observed that treating the cells with increasing concentrations of thymoquinone (0–100 µM) led to a dose and/or time-dependent reduction in cell viability (Figure 1A). The corresponding IC_50_ values for HCT116 and SW480 at 24 h (post-treatment) were 21.71 µM and 20.53 µM, and those at 48 h (post-treatment) were 10.26 µM and 10.50 µM, respectively, (Figure 1A,B). The lowest:highest percent death induction in HCT116 and SW480 at 24 h (post-treatment) was 20.35 ± 4.01; 74.95 ± 5.39 and 17.71 ± 3.87; 76.67 ± 4.16 while at 48 h (post-treatment) it was 44.87 ± 4.38; 88.30 ± 6.26 and 48.04 ± 4.47; 87.13 ± 6.18, respectively. We also evaluated cell death over a time period of 1–4 days by employing a trypan blue dye exclusion assay; the results were consistent with MTT results showing more cell death/inhibition in cell doublings per day with the increase in drug concentration (Figure 1B). We did not evaluate TQ’s biocompatibility on normal intestinal cells because the same was previously reported to be well tolerated by normal intestinal cells for concentrations of up to 60 µM [33]. 

Since uncontrolled cell proliferation is a well-established cancer hallmark [2], we next attempted to evaluate whether sub-lethal dose/s of TQ have any modulatory effect on the proliferation index of CRC cells. It was observed that pre-treatment of TQ in both HCT116 (21.71 µM) as well as SW480 (20.53 µM) cells for 24 h led to a significant reduction in proliferation rate over time (0–4 days) (Figure 1C), highlighting its potential anti-proliferative role against CRC. 

### 2.2. Thymoquinone Modulates Glycolytic Metabolism in CRC Cells

**Figure 2:**
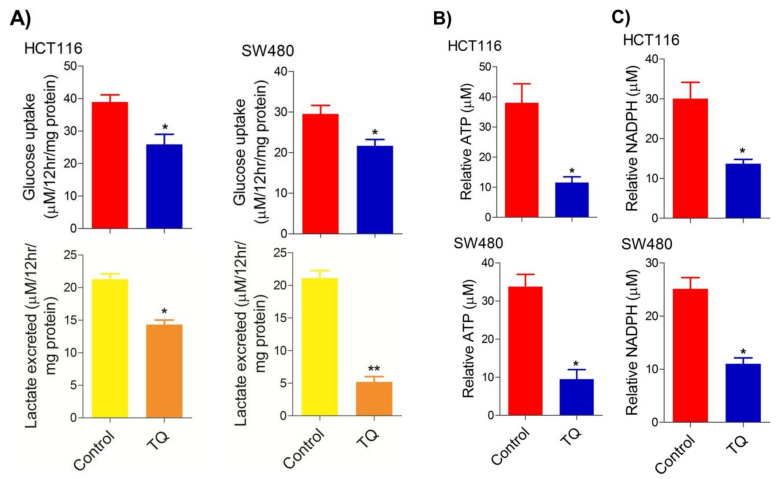
Thymoquinone reduces glycolytic metabolism in CRC. HCT116 and SW480 CRC cells were treated with thymoquinone as indicated. Thymoquinone treatment led to a significant reduction in—(**A**) glucose fermentation rate/s (glucose production and/or lactate production), (**B**) ATP production, and (**C**) redox state (NADPH) in these cells. Statistical significance was calculated by employing unpaired t-test, data expressed as mean ± SEM (*n* = 3). * *p* < 0.05 and ** *p* < 0.01.

Deregulated cellular metabolism is a characteristic feature of most, if not all, cancerous cells and an emerging cancer hallmark [34,35], which expedites the cell-autonomous proliferation and survival of these cells [36]. The Warburg effect manifests an aberrant metabolic state in which cancer cells ferment glucose at higher rates to sustain the higher energy needs as well as biomass production, and hence ensure increased proliferation [5,37]. To this end, we speculated on studying whether cell death induced by TQ in CRC cells (HCT116 and SW480) could be attributed to the inhibition of the glycolytic phenotype in these cells. We found that sub-lethal doses of TQ in both HCT116 (21.71 µM) and SW480 (20.53 µM) after 24 h of treatment significantly reduced the glucose fermentation potential of these cells (Figure 2A). Additionally, such reduction in glucose fermentation capacity also led to a significant reduction in overall ATP production as well as maintaining the redox state (NADPH production) of these cells (Figure 2B,C). These results demonstrate that TQ-induced cell death and/or proliferation inhibition in CRC could be attributed to modulation of the glucose metabolic reprogramming, otherwise necessary to ensure increased biomass production and maintaining redox balance for sustaining proliferative signaling. 

### 2.3. Thymoquinone Inhibits Hexokinase 2 via Modulating the PI3K-AKT Pathway

**Figure 3:**
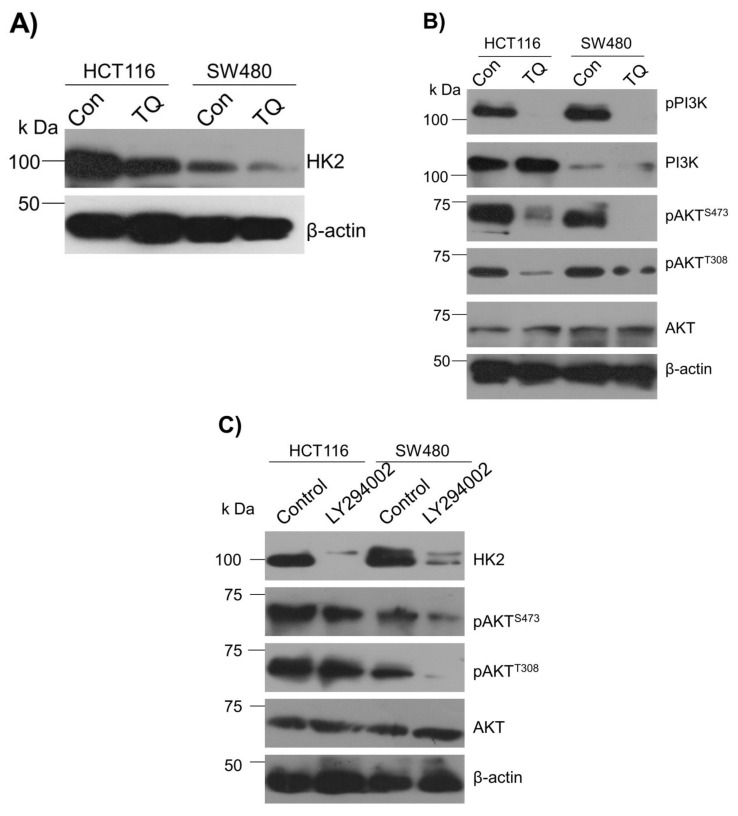
Thymoquinone inhibits Hexokinase-2 via modulating the PI3K-AKT pathway. HCT116 and SW480 CRC cells were treated with thymoquinone or LY294002 as indicated. (**A**) Thymoquinone treatment led to inhibition in HK2 protein levels. Such inhibition in HK2 was concomitant to inhibition in—(**B**) the PI3K-AKT pathway. (**C**) Inhibiting the PI3K-AKT pathway using selective PI3K pharmacologic inhibitor LY294002 led to an inhibition of HK2 protein levels.

To underpin the role behind the reduction in the glucose fermentation rate in TQ-treated CRC cells, we evaluated the expression status of HK2, the first rate-limiting enzyme of glucose metabolism and an enzyme highly overexpressed in most cancers including CRC [38,39,40,41]. It was observed that TQ treatment substantially inhibited HK2 expression in HCT116 and SW480 cells (Figure 3A). Further, to know the mechanism behind the HK2 reduction, we evaluated the PI3K-AKT levels because previously it was reported that PI3K-AKT induces glycolytic phenotype via HK2 in pediatric osteosarcomas [13]. Expectedly, the reduction in HK2 levels upon TQ treatment coincided with significant inhibition in PI3K-AKT activation (i.e., reduction in p-PI3K-p-AKT^T308/S473^) (Figure 3B), suggesting that TQ inhibits HK2 via modulating the PI3K-AKT pathway. To confirm whether such reduction in HK2 was PI3K-AKT-dependent, we used the selective pharmacologic inhibitor (LY294002) of PI3K and we observed a significant reduction in HK2 levels concomitant to PI3K-AKT inhibition (Figure 3C). These results demonstrate that TQ inhibits glycolytic metabolism in CRC cells by inhibiting HK2 via the PI3K-AKT pathway.

### 2.4. Inhibition of the PI3K-AKT/ HK2 Pathway Abrogates the Tumorigenic Potential of CRC Cells

**Figure 4:**
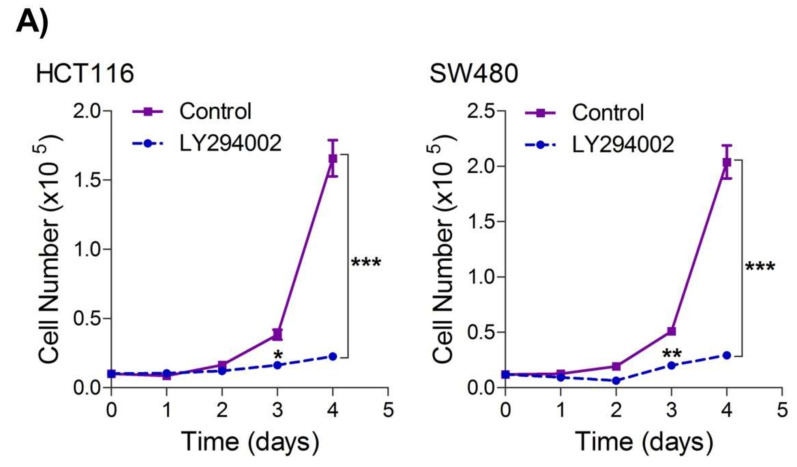

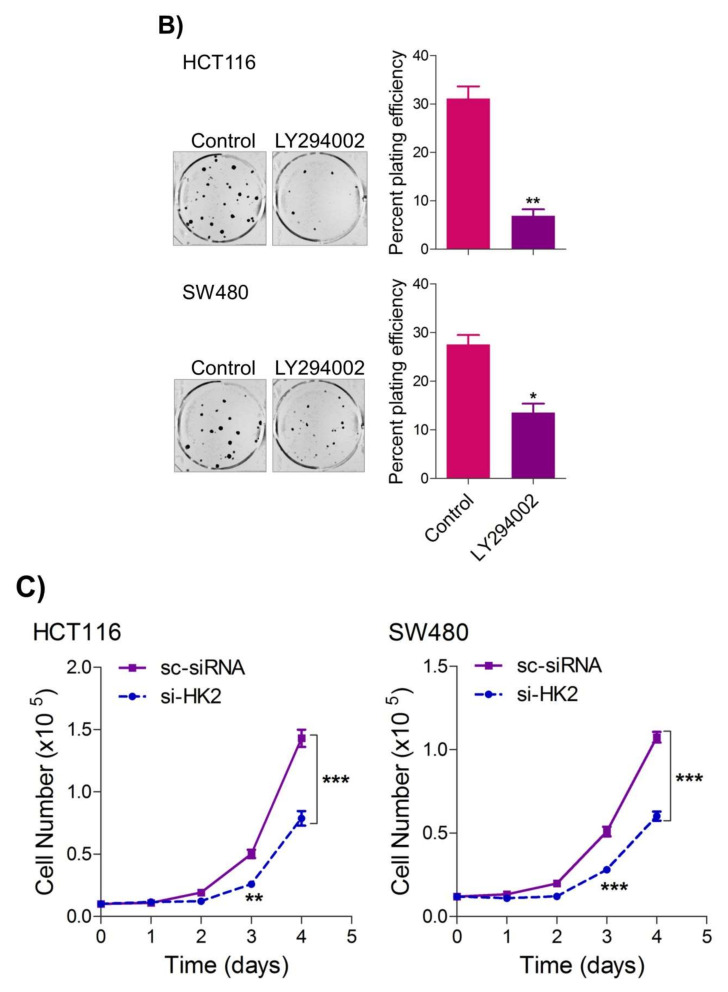

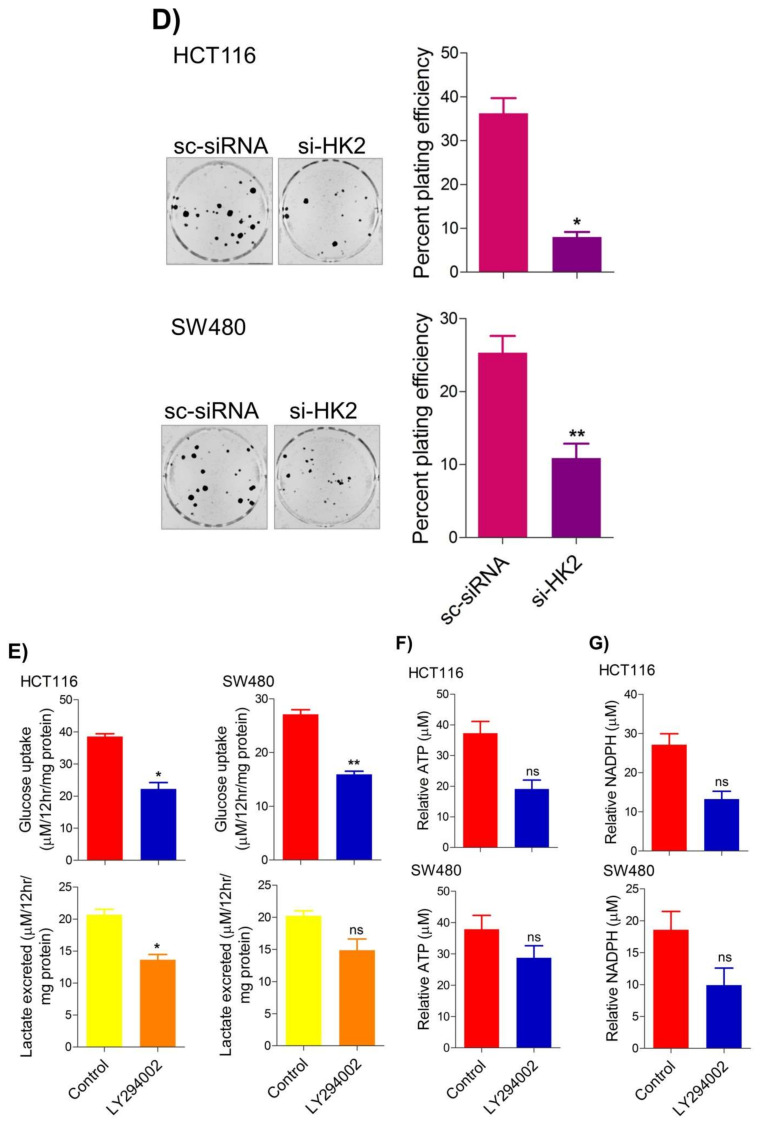

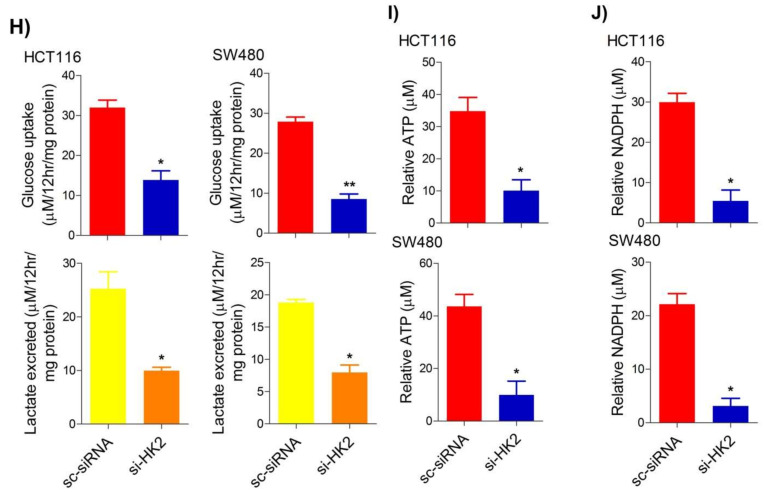
Inhibition of PI3K-AKT/ HK2 pathway impedes tumorigenic potential of CRC cells. HCT116 and SW480 CRC cells were treated as indicated. Inhibition of PI3K with its pharmacologic inhibitor LY294002 led to a significant inhibition in—(**A**) cell proliferation, and (**B**) clonogenic propensity of these cells. Genetic (siRNA) or pharmacologic (3-Bromopyruvate) inhibition of HK2 led to a significant reduction in—(**C**) proliferation, and (**D**) Clonogenic potential of these cells. Statistical analyses were carried out employing two-tailed paired *t*-test (proliferation assays) and two-way ANOVA-(Clonogenic assays) followed by appropriate post hoc tests (Bonferroni). Pharmacologic (LY294002) inhibition of PI3K led to an inhibition in (**E**) glucose fermentation rate/s (glucose uptake and lactate excretion), (**F**) ATP production and (**G**) redox state (NADPH). Genetic (siRNA) ablation of HK2 led to a significant abrogation in (**H**) glucose fermentation rate/s (glucose uptake and lactate excretion), (**I**) ATP production and (**J**) redox state (NADPH). Statistical significance was calculated by employing unpaired t-test, data expressed as mean ± SEM (*n* = 3 or 4). * *p* < 0.05, ** *p* < 0.01, *** *p* < 0.001.

To seek whether targeting the PI3K-AKT/HK2 pathway also abrogates the tumorigenic propensity of CRC cells, we used a selective pharmacologic inhibitor of PI3K (LY294002) to study colony formation and cell proliferation over time. It was found that PI3K inhibition significantly inhibited colony formation potential as well as proliferation over time (Figure 4A,B); further confirming that the carcinogenic propensity of CRC cells (HCT116 and SW480) is majorly regulated via the PI3K-AKT axis. More importantly, genetic (siRNA) and low-dose pharmacologic (10 µM, 3-Bromopyruvate) ablation of HK2 led to a significant reduction in cell proliferation as well as colony formation propensity in these cells (Figure 4C,D). Additionally, pharmacologic silencing of PI3K also led to a significant rescue in glycolytic reprogramming (Figure 4E–G), similarly, genetic ablation (siRNA-mediated) of HK2 also showed a significant reduction in glucose fermentation, ATP and NADPH production rates (Figure 4H–J). These results demonstrate that PI3K-AKT/HK2 activity directly regulates the tumorigenic propensity of CRC cells, which can be effectively targeted by TQ.

### 2.5. Thymoquinone Inhibits Cell Migration and Invasion via Modulating Glucose Metabolic Reprogramming

**Figure 5:**
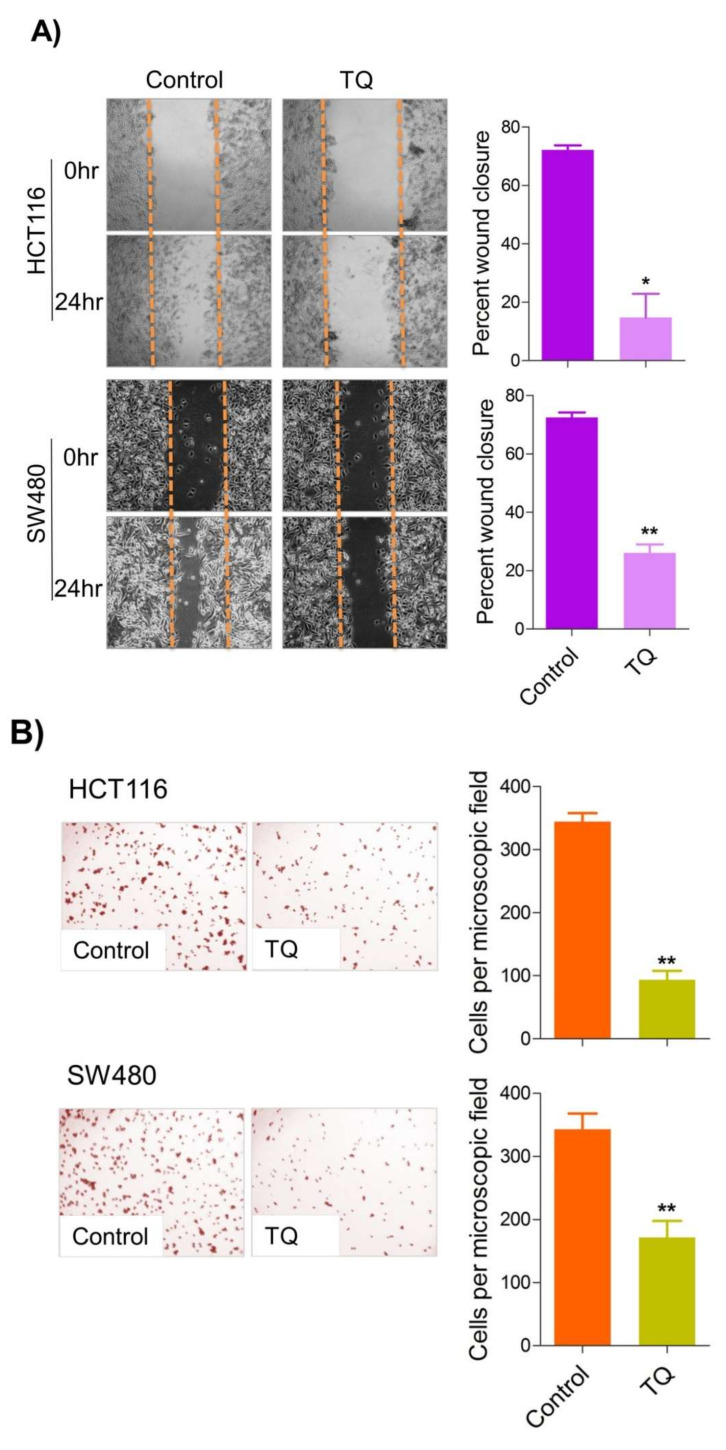

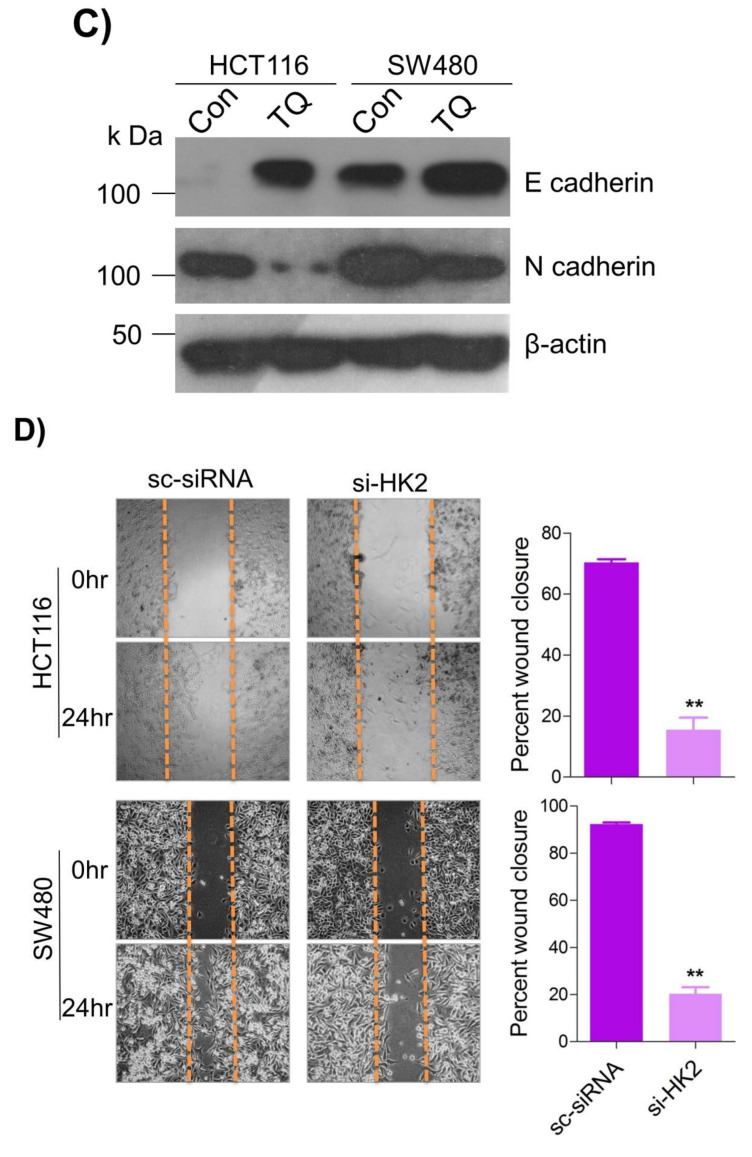

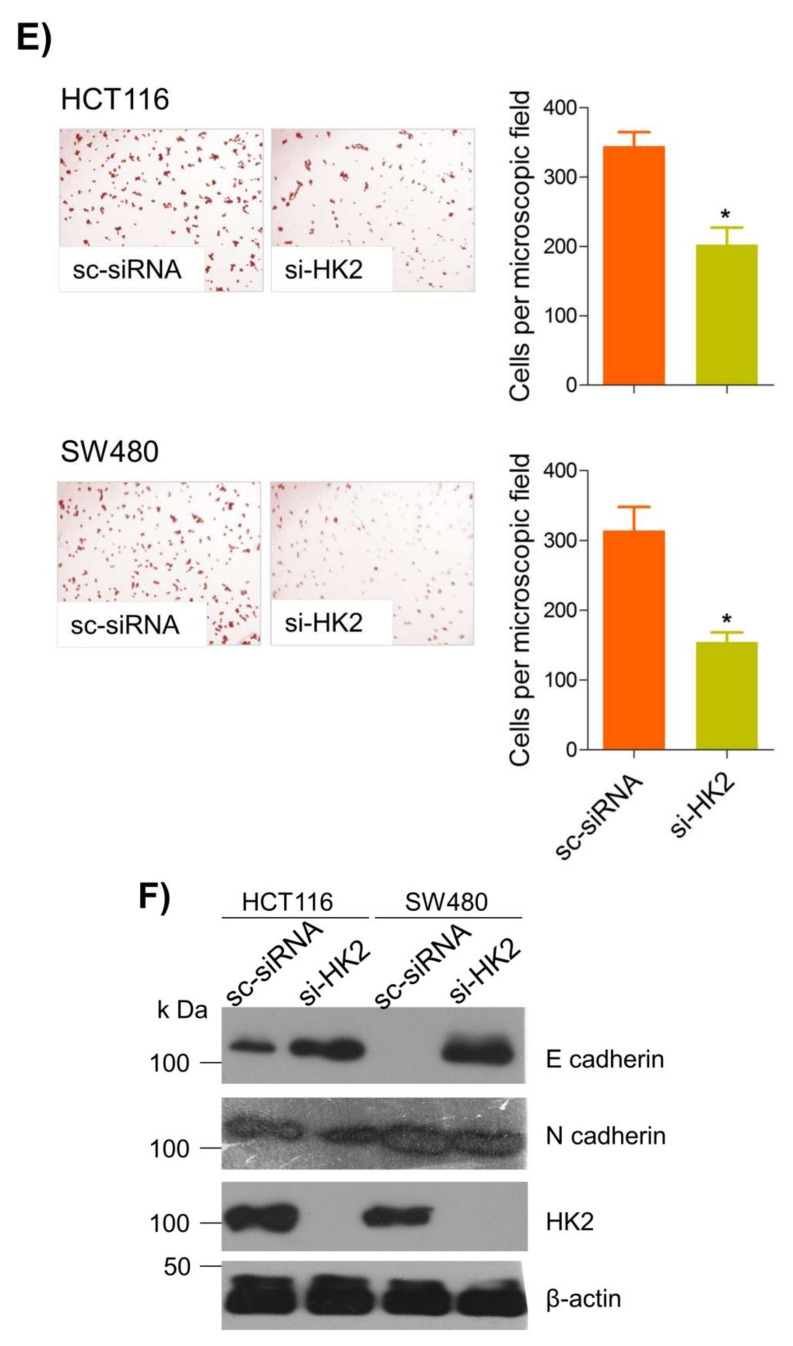
Thymoquinone inhibits cell migration and invasion via modulating glucose metabolic reprogramming. HCT116 and SW480 CRC cells were treated as indicated. Treating these cells with thymoquinone led to a significant inhibition in—(**A**) wound healing, and (**B**) invasion potential. (**C**) Thymoquinone also led to induction in E cadherin with a concomitant inhibition in N cadherin levels. Genetic (siRNA) ablation of HK2 led to a significant inhibition in—(**D**) wound healing, and (**E**) cell invasion. (**F**) HK2 ablation also led to induction in E cadherin and inhibition in N cadherin levels. Statistical analyses were carried out by employing two-tailed paired *t*-test, data expressed as mean ± SEM (*n* = 3). * *p* < 0.05, ** *p* < 0.01.

Metastatic dissemination is one of the potent hallmarks of cancer [2], and with glucose metabolic reprogramming having a strong role in metastatic dissemination [9,42], our next interest was to seek whether and how thymoquinone impacts metastasis in CRC cells. To this end, we found that TQ treatment significantly inhibited the wound healing propensity and invasiveness of HCT116 and SW480 cells (Figure 5A,B), indicating its anti-metastatic potential. At the molecular level, TQ was able to induce E-cadherin while inhibiting N-cadherin expression (Figure 5C). Further, to confirm whether such inhibition in metastatic propensity was through glucose metabolic reprogramming, we employed genetic ablation of HK2 to inhibit the glucose uptake and hence glycolysis, which was found to rescue both wound healing as well as invasiveness in HCT116 and SW480 cells (Figure 5D,E). At the molecular level, such inhibition in glycolysis led to the induction of E-cadherin with a concomitant inhibition in N-cadherin levels (Figure 5F). Taken together, these data suggest that TQ inhibits metastatic predisposition of CRC cells, possibly via modulation of glucose metabolic reprogramming.

## 3. Discussion

Though a substantial advancement has been achieved in CRC treatment due to scientific advancements in the past 10–15 years, it still remains one of the major causes of deaths due to cancer globally [43,44], besides being the most prevalent disease in the young (below 50 years of age) in more advanced stages [45]. No doubt many efforts are underway towards improving the systemic treatment of patients with metastatic colorectal cancer (m-CRC). Glucose metabolic reprogramming along with the aberrant tumor microenvironment (TME) has posed a challenge to effectively target the disease progression [46]. The “Warburg effect” or “aerobic glycolysis”, which is considered an important metabolic driver in the development of most solid cancers including CRC, leads to perturbations in the way cells consume nutrients to drive metabolic pathways [5,47]. Such aberrant glucose uptake and lactate production ensure higher anabolic needs of aberrantly proliferating CRC cells besides acidifying the tumor microenvironment (TME) to induce angiogenesis [5,48], metastatic dissemination [49,50], chemotherapeutic resistance [51,52] and immune evasion [53,54,55]. Such aberrant metabolic signatures not only provide growth advantages but also offers a therapeutic modality to selectively target such abnormal cells [56,57]. In this regard, various antimetabolite drugs were discovered that have shown a significant reduction in tumor burden by selectively targeting the aberrantly behaving metabolic enzymes/pathways [58,59,60,61,62,63,64,65,66], and many are undergoing clinical trials. Many phyto-compounds were reported to inhibit cancer growth [67,68] and cancer cell metabolism [8,69], highlighting the need to screen and study more phyto-compounds against cancer. To this end, we evaluated if and how TQ, a well-studied anticancer [70,71] (reviewed in [72]) secondary plant metabolite, modulates glycolytic phenotype in CRC cells. We found that TQ inhibits cell proliferation, clonogenicity and epithelial-mesenchymal transition (EMT) in CRC cells (HCT116 and SW480) and such inhibition is attributed to its role in modulating glycolytic metabolism of these cells. Mechanistically, TQ’s anti-carcinogenic potential is attributed to its inhibitory effects on the rate-limiting glycolytic enzyme HK2, leading to a reduction in glucose fermentation rate and hence inducing energy debt and dis-balancing the redox state of the proliferating CRC cells. Interestingly, such an anti-glycolytic effect was regulated at least in part via the PI3K-AKT pathway, the most aberrated pathway in cancer [12]. 

In human beings, HKs are a group of four (HK1-4) tissue-specific iso-enzymes that catalyze the first step (phosphorylation of glucose) in glucose metabolism [73]. However, the HK2 isoform is the critical regulator of the Warburg effect in many cancer types, viz prostate, hepatocellular, gliomas [39], breast [41], gastrointestinal [74], lung [63] and ovarian cancers [40,75], making it an important therapeutic target against cancer. TQ showed concentration-dependent cell death in HCT116 and SW480 cells (Figure 1), showing its potential as a cytotoxic agent capable of inhibiting cell proliferation in CRC. Such anticancer effects of TQ have previously been reported in various cancer models including CRC [24,76,77,78,79,80] with various modes of action. Though TQ is reported to induce cell death in renal cell carcinoma [81] and pancreatic cancers [82] via inhibiting HIF1α and pyruvate kinase M2 (PKM2)-mediated glycolysis, further studies are warranted to elucidate the underlying mechanism/s using more cell models. Our results show for the first time that TQ-mediated proliferation arrest in CRC cells is attributed to its negative impact on the Warburg effect, otherwise necessary for the growth and proliferation of cancerous cells including CRC [5,9]. TQ treatment inhibited the glucose uptake and subsequent lactate production in HCT116 and SW480 cells, inducing a net energy debt and inhibiting the anabolic metabolism, otherwise necessary to support macromolecule biosynthesis of highly proliferating cells. The decrease in HK2 expression levels explains the regulatory knot behind such inhibition of glucose fermentation, post-TQ treatment. The reduction in cell proliferation and/or clonogenicity, besides a rescue in glucose fermentation rate upon HK2 silencing (Figure 2), explains that such tumorigenic propensity in CRC cells is dependent on aberrant metabolic nature regulated, at least in part, via HK2. 

The PI3K-AKT pathway is the most aberrant pathway in many cancers [12,83] including CRC (reviewed in [84,85]), which not only activates cell proliferation, growth metastasis but also induces apoptotic evasion. The PI3K-AKT was established to positively regulate the Warburg effect via increased glucose uptake as well as phosphorylation and activation of glycolytic enzymes (reviewed in [36]). Moreover, PI3K-AKT was also reported to induce the Warburg effect via up-regulating HK2 expression and tumorigenic growth in osteosarcoma [13]. Our results also confirmed that aberrant glucose fermentation rate and enhanced tumorigenicity in CRC cells may be attributed to activating the PI3K-AKT/HK2 axis besides some other oncogenic events [9], evidenced by direct inhibition of HK2 expression and tumorigenicity upon selectively inhibiting PI3K-AKT. In corroboration with our findings, constitutively active AKT was found to sufficiently trigger glycolysis [86,87], leading to increased uptake of glucose and glycolytic flux independent of growth factor signaling [86,87,88,89,90,91], thus rendering such cells to rely on glucose to survive [87,91]. To the best of our knowledge, results from the present study demonstrate for the first time that TQ induces proliferation arrest and cell death in CRC by inhibiting PI3K-AKT, which in turn inhibits HK2 and hence glucose uptake, subsequently leading to the ATP debt and inhibiting molecular building blocks otherwise necessary for continued proliferation and growth of cancerous cells [56,92]. More importantly, the reduction in the NADPH levels upon TQ treatment indicates an inhibition in reductive biosynthesis while inducing oxidative stress [36,93,94,95]. In similar lines, PI3K-AKT was known to regulate multiple checkpoints in the glycolytic pathway, viz post-translational as well as transcriptional gears on glucose transporter and/or other glycolytic enzymes [36]. 

**Figure 6:**
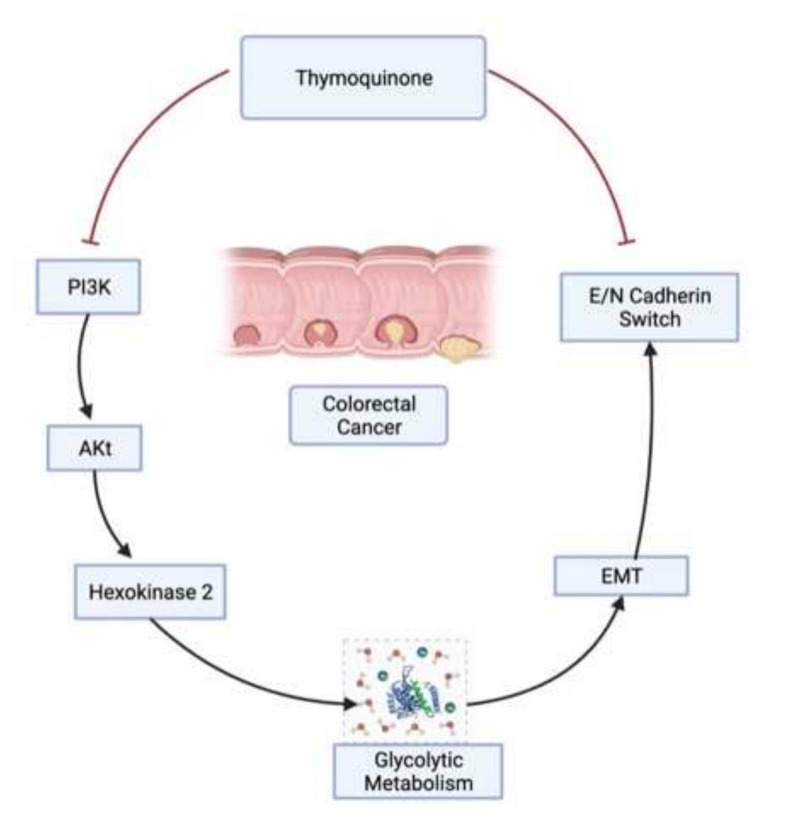
Overall mechanistic representation of thymoquinone in colorectal cancer.

Metastasis is a multistep process that is regulated by multiple factors and accounts for approximately 90% of cancer-related mortality across the world [96]. Epithelial-mesenchymal transition (EMT), an early metastasis step, is known for the dissemination of circulatory tumor cells (CTC’s) into the bloodstream and accounts for the worse cancer prognosis [97,98,99]. Moreover, approximately 20% of newly detected CRC cancers are metastatic and about 20% more will transform into metastatic, accounting for lower survival rates [100,101]. In the present study, we found that TQ treatment to HCT116 and SW480 cells led to inhibition in cell invasion and concomitant induction of epithelial cell marker E-cadherin while down-regulating the mesenchymal marker N-cadherin. Furthermore, TQ treatment also abrogated the wound healing potential of these cells, further supporting its anti-metastatic effect of TQ [102,103]. Though TQ has previously been shown to show strong anti-metastatic effects [102,103], the mechanisms are still not very clear. The results from the present study provide evidence that anti-metastatic effects of TQ are mediated by a reduction in the glycolytic rate [9,104,105,106], which in turn modulates redox status and induces an energy crisis otherwise necessary for metastasizing cells. Such metabolic intervention by TQ was further confirmed upon inhibiting HK2-mediated phosphorylation of glucose (using the 3-bromopyruvate/ siRNA approach). Overall, the results from the present study underpin a novel mechanism of TQ against CRC progression by a reduction in the glycolytic rate, possibly mediated via the PI3K-AKT/HK2 axis (Figure 6). We suggest employing other cancers and/or employing suitable in vivo models in more advanced settings to decipher such anticancer effects of TQ. 

## 4. Materials and Methods

### 4.1. Cell Lines

The study was conducted using two CRC cell lines, HCT116 and SW480, acquired from National Centre for Cell Sciences, Pune India (NCCS, Pune) India. Both the cell lines were grown in Dulbecco’s Modified Eagles Medium (DMEM, Gibco) supplemented with 2-mM glutamine, 1× pen/strep (Gibco) and 10% Fetal Bovine Serum (FBS, Gibco) at 37 °C temperature and 5% CO_2_ under humid settings in a cell culture incubator (Shell labs).

### 4.2. Cytotoxicity (MTT) and Cell Doubling Assays

MTT colorimetric test with thymoquinone was performed in CRC cell lines (HCT116 and SW480). For 24 h, we seeded 3 × 10^4^ HCT116 and SW480 in each well of a 96-well plate. The cells were subsequently incubated with increasing concentrations of thymoquinone (Selleckchem, Cat no.S4761) (0–100 µM). Following a 24-h incubation period, the treated samples were withdrawn from the wells and the cells were incubated for 4 h at 37 °C with 25 L of MTT solution (0.5%, *w*/*v*). The unreacted MTT solution was withdrawn after incubation, and the cells were exposed to 150 L DMSO (Selleckchem, Cat no. B14001) in each well for 20 min to solubilize the produced formazan crystals. Finally, using a microplate reader (Thermo Fisher Scientific, Waltham, MA, USA), the absorbance of the formazan solution in each well was measured at 570 nm; the absorbance was directly proportional to the number of viable cells in the wells.

The assay was performed to study the effect of TQ on cell doubling, as per previously described method with little modifications [107]. Briefly, the cells (1.0 × 10^4^) cells were seeded in 12 well plates in triplicate and allowed to attach overnight. Next day the cells from one representative well were trypsinized to count them to get initial cell number, and rest wells treated with TQ and allowed to incubate for 4 days. The cells were counted after 4 days post-treatment, using Neubaur Chamber to calculate cell doubling as:

Proliferation Rate (Doubling/day) = Log_2_ (Final cell count (day 5)/cell count (day 1)/4 (days)).

### 4.3. Colony Formation Assay

The colony formation assay was performed to study the effect of indicated treatments/ genetic modifications on cologenic potential of CRC cells, following the previously defined protocol with slight modification [9]. Briefly, the cells were either treated with low dose pharmacologic inhibitor of PI3K (LY294002) for 48 h at which time the cells were trypsinized at seeded at a density of 200 cells/ 6-well in triplicate and allowed to grow for 3–4 weeks until individual colonies became visible. Then the media was removed out and cells were stained with 0.1% crystal violet for half an hour, followed by gentle rinsing in still tap water until excess dye was washed out. After washing, the plates were scanned on white background using Chemodoc XRS+ from Bio Rad to count the visible cell colonies in different groups and calculate the plating efficiency. Plating efficiency was similarly performed post HK2 silencing using siRNA gene silencing approach.

### 4.4. Cell Proliferation Assay

The low passage healthy cells were seeded in 60 mm dishes at 6.5–7.5 × 10^5^ cell density and treated next day with IC_50_ concentration of TQ or low dose LY294002 (PI3K inhibitor) for 36 h at which time the cells were trypsinized and seeded in 12 well plates at a density ranging from 1.0 × 10^4^–1.5 × 10^4^ cells per well in 4 replicates in quadruplet (1–4). Each day, a couple of wells from each treatment group were trypsinized and counted manually; using a Neubauer chamber and a growth curve was generated over a period of 96 h. For HK2 inhibition, siRNA transfected cells along with corresponding mocks (sc-siRNA groups) were seeded after 40–48 h post-transfection and the proliferation evaluated as above. 

### 4.5. Colony Formation Assay

#### siRNA Transfection

siRNA transfections were given transiently for control siRNA (sc-siRNA), sense-UUCUCCGAACGUGUCACGUTT anti sense-ACGUGACACGUUCGGAGAATT oligos (IDT), sequences described previously [108], and predesigned silencer select HK2 siRNA, Thermo (ID:144506; AM16708) using Lipofectamine RNAi max reagent (Thermo scientific) as per manufacturer instructions following our previously reported procedure with slight modifications [9]. Briefly, the cells at around 70% confluence transfected with 20 nM of indicated Oligos after serum starving them for 4–5 h. The transfected cells were replenished with fresh media after 8 h and gene silencing was assessed after 72 h, post-transfection using Western blot. For cell proliferation, clonogenic, metabolic and/or metastasis-related assays the transfected cells were trypsinized at 48 h (post-transfection) and seeded at appropriate densities into 12 well or 35 mm dishes as needed.

### 4.6. Wound Healing and Cell Invasion Assays

The wound-healing assay was carried out on HCT116 and SW480 cells for evaluating the cell migration, following previously described procedure with some modifications [9]. Briefly 1.5–2 × 10^5^ cells were seeded in 12 well plates in triplicate and allowed to grow to confluency (approx. >90%). After this, the cells were serum-starved for 7–8 h and scratches were made using a P10 tip, along the respective diameter of the wells. Then the media was gently aspirated and the detached cells washed off using 1× PBS. The cells were then replenished with low serum (0.5%) cell culture media or low serum media along with 25 μM TQ or 20 μM 3-bromopyruvate (3-BP) as indicated. Images were taken at 0 and 24 h after scratch was made and the migratory propensity calculated in terms of wound closure in respective groups using Image J (NIH) software. Further, the cell invasion assay/s was carried out using transwell inserts, for which about 1 × 10^5^ cells were seeded in serum-free media (200 μL) or 25 μM TQ/20 μM 3-BP in 24 well transwell inserts (SPL Insert; catalog 36224), coated with 40 μL extracellular matrix (ECM). The complete growth media (600–700 μL) containing 10% FBS was put in the lower chamber of the plate to serve as chemo-attractant and induce cells to pass across transwell pores. The cells were pretreated with indicated drugs (TQ or 3-BP) for 24 h before carrying out the cell invasion assay/s. Similarly, pre-transfected (48 h before) si-HK2 groups were processed in the same way as above. After 24 h of incubation, the inserts were washed softly using 1X PBS, fixed in ice-cold ethanol (70%) for 15 min and then stained using 0.1% crystal violet dye for next 10–15 min. Finally, after de-staining in tape water, the wells were swabbed from inside to remove matrigel and noninvasive cells. Invasion efficiency was calculated by counting the cells in random fields, under microscope and representative images taken.

### 4.7. Western Blot

The indicated cell groups were trypsinized and lysed in Pierce IP lysis buffer (Thermo Fisher Scientific) containing 0.5% sodium deoxycholate, 0.1% SDS and 1X Sigma fast (Sigma Aldrich) for 10–15 min on ice. The lysate was centrifuged at 10,000 rpm for 10 min at 4 °C. The supernatant was aspirated into fresh Eppendorf tubes and total protein quantified using BCA kit as per manufacturer instructions (Thermo Scientific). Equal amounts of protein (40–50 μg) were resolved on SDS PAGE and transferred onto PVDF membrane (Bio Rad) employing wet transfer at 75 V for 1.5 h at 4 °C. The membranes were probed with indicated primary antibodies overnight at 4 °C after blocking them in 5% skimmed milk (in 1× TBST) for 1 h. After 12 h of primary probing, the membranes were washed thrice for 5 min each in 1x TBST followed by secondary probing with appropriate HRP tagged secondary antibodies at room temperature for 1 h. The protein expression was observed using chemiluminescence-based detection on X-ray film (Amersham). The primary antibodies used were against β-actin (NB600-501, Novus Biologicals), E-cadherin (NBP2-19051, Novus Biologicals), N-cadherin (NBP1-48309, Novus Biologicals), HK2 (STJ99121, Saint Johns), pAKT^S473^ (sc-293125, Santa Cruz), pAKT^T308^ (sc-135650, Santa Cruz), pPI3K (4228S, Santa Cruz) and the corresponding secondary antibodies used were either goat anti-rabbit IgG-HRP (NB7160, Novus Biologicals) or goat anti-mouse IgG-HRP (NB7539, Novus Biologicals). 

### 4.8. Glucose Uptake/Lactate Production, ATP, and NADPH Measurement

Glucose uptake and lactate production was measured in the spent media using respective kits (Eton Bioscience, SKU-1200032002 and 1200012002) as per manufacturer instructions. Similarly, ATP levels were measured using ATP Colorimetric/ Fluorometric Kit (Bio vision, K354-100) and NADPH levels were quantified using Ampelite Colorimetric NADPH Assay Kit (AAT Bioquest, 15272) as per manufacturer instructions. 

### 4.9. Statistical Analyses

The statistical analyses were carried out using Graph Pad Prism V 5.0. The differences between groups were analyzed either by unpaired *t*-test or two-way ANOVA as applicable with appropriate post hoc tests as mentioned. All the data are represented as mean ± SEM of at least three replicates unless otherwise stated. *p* values corresponding to *p* ≤ 0.05 were considered statistically significant.

## Data Availability

Not applicable.
